# Hollow Fiber Membrane Dehumidification Device for Air Conditioning System

**DOI:** 10.3390/membranes5040722

**Published:** 2015-11-16

**Authors:** Baiwang Zhao, Na Peng, Canzeng Liang, Wai Fen Yong, Tai-Shung Chung

**Affiliations:** Department of Chemical & Biomolecular Engineering, National University of Singapore, Singapore 117585, Singapore; E-Mails: chezhba@nus.edu.sg (B.Z.); pengna1029@hotmail.com (N.P.); chelian@nus.edu.sg (C.L.); cheywf@nus.edu.sg (W.F.Y.)

**Keywords:** hollow fiber, module fabrication, PDMS coating, membrane dehumidification, energy saving

## Abstract

In order to provide a comfortable living and working environment indoors in tropical countries, the outdoor air often needs to be cooled and dehumidified before it enters the rooms. Membrane separation is an emerging technology for air dehumidification and it is based on the solution diffusion mechanism. Water molecules are preferentially permeating through the membranes due to its smaller kinetic diameter and higher condensability than the other gases. Compared to other dehumidification technologies such as direct cooling or desiccation, there is no phase transition involved in membrane dehumidification, neither the contact between the fresh air stream and the desiccants. Hence, membrane dehumidification would not only require less energy consumption but also avoid cross-contamination problems. A pilot scale air dehumidification system is built in this study which comprises nine pieces of one-inch PAN/PDMS hollow fiber membrane modules. A 150 h long-term test shows that the membrane modules has good water vapor transport properties by using a low vacuum force of only 0.78 bar absolute pressure at the lumen side. The water vapor concentration of the feed humid air decreases dramatically from a range of 18–22 g/m^3^ to a range of 13.5–18.3 g/m^3^. Most importantly, the total energy saving is up to 26.2% compared with the conventional air conditioning process.

## 1. Introduction

Singapore as one of the tropical countries and it has a uniformly high temperature from 28 °C to 32 °C and a humidity of above 85% throughout the year [1]. However, the recommended indoor air temperature and relative humidity for living and working comfortably is around 23 °C to 26 °C and 45% to 60%, respectively [2]. In order to provide a comfortable living and working environment indoors, specifically in Singapore, the outdoor air should be cooled and dehumidified before it is circulated into the rooms. The current widely used and accepted technologies for removing air moisture are either direct cooling by chillers or desiccations. As for the direct cooling, the chiller in the air handling unit (AHU) usually first cools the humid air to its saturation temperature and then condenses the excessive moisture into water. Since the moisture goes through the phase transition process during the condensation, direct cooling is energy intensive. Removing humidity by desiccants is a simple method [3,4,5,6]. However, the process to regenerate the desiccants is energy intensive and desiccants have the risk of cross-contamination to the air stream.

Membrane technology for air dehumidification is based on the solution diffusion mechanism, where the water molecule preferentially permeates through the membranes due to its smaller kinetic diameter and higher condensability than the other gases. There is no phase transition involved neither the contact between the fresh air stream and the desiccants, hence it requires minimal energy consumption and can avoid the cross-contamination problems. In addition, membrane systems have the advantages of a small footprint, and easy scale-up and adaption compared to other available units in the whole system.

Since the selectivity of polymeric materials for water over air is usually high [7], gas separation membranes can be used for dehumidification directly without modifications. From the first success of the Prism’s hydrogen recovery process, followed by the separation of carbon dioxide from methane, oxygen from nitrogen, membrane gas separation technologies have received significant attention from both academia and industries over the years [8,9,10]. To date, about less than 10 polymeric materials have been used in commercial gas separation membranes out of hundreds of polymers synthesized for gas separation studies in the literature [11,12,13,14]. One of the most important criteria for commercially available gas separation membranes is to have an ultra-thin dense selective layer [15,16] to promote high gas transport properties. Both integrally skinned and multilayer composite approaches have been employed to fabricate gas separation membranes [15,16,17,18,19,20,21,22,23,24,25]. For dehumidification membranes, the microporous support layer may be the dominant resistance to water transport, which is opposed to traditional gas separation membranes where the selective layer is the dominant resistance. Thus, a desired membrane comprising a highly microporous substrate and a thin, dense selective layer is crucial for dehumidification system [26].

Peinemann and his co-workers [23,24] and Li *et al*. [25] have successfully fabricated various multi-layer composite membranes with superior CO_2_/N_2_ and O_2_/N_2_ separation performance. Since we are familiar with Li *et al*. approach and their composite PDMS-coated PAN hollow fibers performed well under low pressures (about 15 psi) compared to other gas separation membranes (usually operated under 100 psi and above), PDMS-coated PAN composite hollow fibers will be fabricated and used in our membrane dehumidification system.

Membrane dehumidification for compressed air or natural gas is a well-studied process and several hollow fiber membrane products are commercially available [27,28,29,30,31,32]. Basically, the moisture permeation rate through a membrane is a function of the partial pressure difference of the moisture across the membrane. Since the gas permeation selectivity of water over air is high, the water vapor partial pressure in the permeate side can quickly reach that of the feed side, as a result, it slows down the dehydration efficiency. One method to overcome it is to utilize a sweep gas [26,33]. Morgan *et al.* from Air Products patented a hollow fiber dehydration method and apparatus using an internal sweep of the permeate side by a dry gas product [7]. This invention works well under high operation pressures (above 7 bars). Other than that, some researchers have studied the impact of vacuum and sweep gas for the feed gas operated below 7 bar and modeling the energy demand for air dehumidification *versus* traditional systems [34,35,36,37].

In this work, we aimed to develop a pilot-scale membrane dehumidifier and pave the feasibility of assembling the membrane system with the AHU units where low pressures are used. Our main objectives are (1) to scale up the PAN/PDMS composite hollow fiber modules from a lab scale to a pilot scale; (2) to design a pilot membrane dehumidification system working under low pressures and test its long-term performance stability; and (3) to calculate the energy savings using our home-made membrane dehumidifier together with the AHU.

## 2. Experimental

### 2.1. Materials

Polyacrylonitrile (PAN) was kindly provided by Prof. Hui-An Tsai from Chung Yun Christian University. N-methyl pyrrolidone (NMP) was purchased from Merck (Singapore). Cyclohexane was from Sigma-Aldrich (Singapore). Gases including O_2_, N_2_, and CO_2_ with purities higher than 99.99% were from Singapore Oxygen Air Liquide Pte Ltd. (SOXAL). The Sylgards^®^ 184 silicone elastomer kit including the silicone elastomer and the curing agent was purchased from Dow Corning Singapore Pte. Ltd. (Singapore).

### 2.2. Preparation of PAN Hollow Fiber Substrates

PAN powder was first dried in a vacuum oven at 50 °C over-night to remove the moisture. After drying, 20 wt % PAN was dissolved in NMP at 50 °C until a homogeneous solution was formed. The PAN single layer hollow fiber membrane was prepared by a dry-jet wet spinning process [38,39]. The spinning conditions are tabulated in Table 1. During the spinning, the dope solution was filtered by a 15 μm metal filter to remove unwanted particles before entering the spinneret. Tap water at room temperature (25 ± 2 °C) was used as the external coagulant and a NMP/water (95/5 wt %) mixture was used as the bore fluid with the purpose of forming a porous inner surface. The nascent fibers underwent an air-gap region with a drawing process to tailor the pore size and pore size distribution of the membranes. After spinning, the nascent hollow fibers were soaked in tap water for two days to remove the remaining NMP. To avoid the collapse of the hollow fiber structure during the drying process, freeze-drying (Freeze-dryer Moudulyod, Thermo Electron Cor., Waltham, MA, USA) at −50°C was utilized [40].

### 2.3. Preparation of the PDMS Coating Solution

A Sylgards^®^ 184 silicone elastomer base was placed in a plastic beaker and preheated to 75 °C. After the temperature was stabilized, a Sylgards^®^ 184 silicone elastomer curing agent was added. The mixture was vigorously stirred by hands. After a certain time, hexane was added into the silicone mixture to dilute it to 15 wt %. The partially cross-linked PDMS solution was kept in a fridge to inhibit further cross-linking reaction. Before use, the PDMS solution was diluted to a preferred concentration using hexane.

### 2.4. Fabrication of PAN-PDMS Composite Hollow Fiber Membranes

The composite hollow fiber membranes were prepared using a dip-coating method. The detailed coating process was described elsewhere [25]. In this work, the PAN hollow fibers were immersed into the 1 wt % PDMS solution for 1 s and dried in air for 48 h to let the PDMS fully cure before module fabrication and dehumidification performance tests.

### 2.5. Single-Filament Module Fabrication and Gas Permeance Tests

Detailed descriptions of module preparation with single fiber and the set-up for gas permeation tests were reported elsewhere [41]. For all the gas permeation tests, the gas flux was measured by applying a pressure of ~15 psig on the shell side and the gas flow rate across the membrane was recorded from the lumen side. The gas permeance and selectivity were calculated using the following equations:

(1) $$\frac{P}{L}=\frac{Q}{A∆P}=\frac{Q}{\pi Dl∆P}$$

(2) α(A/B)=(PL)A(PL)B

where P/L is the gas permeance with a unit of GPU (1 GPU = $10^{6}$ cm^3^ (STP)/(cm^2^ s cmHg)); Q is the gas flow rate (cm^3^/min); D and l are the fiber outer diameter (cm) and length (cm), respectively; ΔP is the pressure(cmHg) difference between the shell side and bore side; and α is the selectivity that is the ratio of the permeances of gases A and B, respectively.

## 3. Hollow Fiber Module Preparation and Dehumidification System

### 3.1. Hollow Fiber Module Preparation

**Figure 1:**
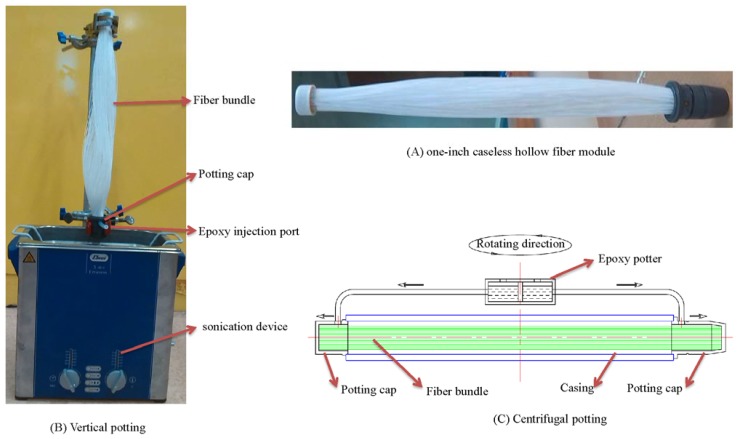
Vertical potting *vs*. centrifugal potting.

To maximize the membrane area but to simplify the design and manufacture process of the membrane dehumidifier, the fibers were potted into one-inch diameter bundles without housings like Figure 1A. Each module bundle contains 400 pieces of PAN/PDMS composite hollow fibers with an effective length of 31.5 cm.

Both vertical potting as in Figure 1B and centrifugal potting as in Figure 1C were used in our experiments. By comparison, the vertical potting process is less dependent on the bulky potting apparatus. In this method, the housing filled with hollow fibers was placed vertically and the potting material was injected from the potting cap via single or multiple channels to balance the flow distribution of the potting material. Only one end of the tube sheet was formed at one time during each vertical potting. A sonication device was employed to assure the homogenous distribution of the epoxy during vertical potting, as well as to minimize the potting defects caused by gas bubbles. Centrifuge potting has to be conducted by a centrifuge potting apparatus which usually contains a rotator which rotates about an essentially vertical axis, at least one housing is mounted on the rotator, one pair of potting caps at the ends of the housing, and containers for the potting materials (as shown in Figure 1C). During the potting process, the selected potting material was delivered to the potting caps by the centrifuge force and tube sheets at both ends of the housing could be formed simultaneously.

### 3.2. Leakage Tests and Repair of the Modules

**Figure 2:**
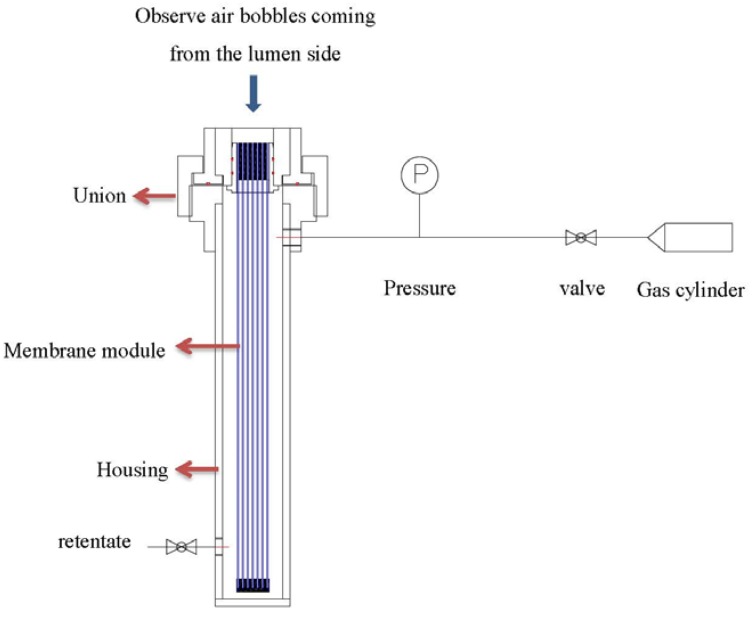
Leaking test of hollow fiber modules.

After potting, the leakage was examined by applying soap water on the cross-section of the module’s lumen while pressurizing from the shell side (as in Figure 2). Bubble generation was observed while air is permeating to the lumen side of the fibers. Locations with obviously faster bubble generation were the indicative of defective regions. The leakages of the modules were sealed by epoxy using a needle syringe.

### 3.3. Pure Gas and Water Vapor Permeance Tests of One-inch Hollow Fiber Modules

**Figure 3:**
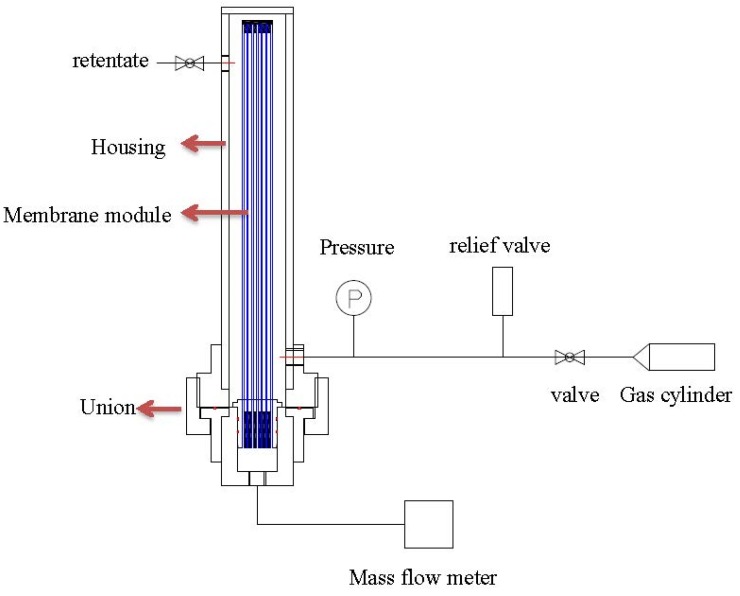
Pure gas test skid of hollow fiber modules.

**Figure 4:**
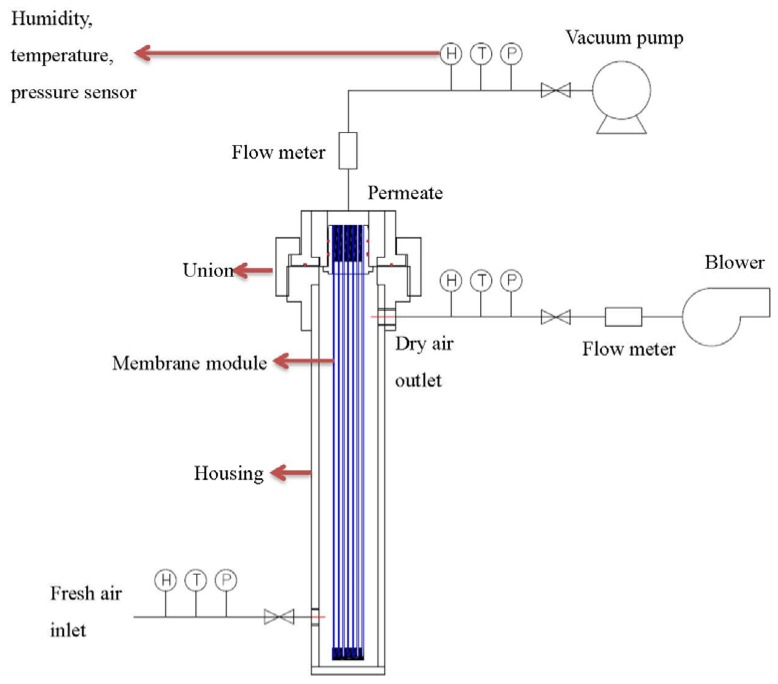
Water vapor test skid of hollow fiber modules.

After the leakage repaired, each of the hollow fiber bundle was tested by pure gases (O_2_, N_2_, and CO_2_) using the setup as shown in Figure 3. The feed gas pressure was about 1 bar while the permeate flow was measured by a mass flow meter. Water vapor permeance tests were carried out with the same setup in Figure 4. Fresh air was fed into the test apparatus using a blower, while the moisture-rich air permeating from the module lumen was removed by a vacuum pump. The humidity, temperature, flow rate and pressure were measured at both the inlet and outlet of the module. With the aid of the psychrometric chart [42], water vapor concentration D (g/m^3^) was obtained from the readings of humidity and temperature. The water vapor flow rate (cm^3^/min) and H_2_O/N_2_ selectivity can be calculated using the following equations:

(3) Q=F(D₁ −D₂ )

(4) α =(PL )H₂O(PL)N₂

where P/L is the water vapor permeance with an unit of GPU (1 GPU = $10^{6}$ cm^3^ (STP)/(cm^2^ s cmHg)); Q is the water vapor flow rate (cm^3^/min); D_1_ and D_2_ are water vapor concentrations (g/m^3^) of inlet and outlet, respectively. F is the humid air flow rate (m^3^/h); α is the selectivity that is the ratio of the permeances of water vapor H_2_O and nitrogen gas N_2_ (pure gas). The water vapor permeance (GPU) can be calculated using Equation (1).

### 3.4. Design of the Membrane Dehumidification System

**Figure 5:**
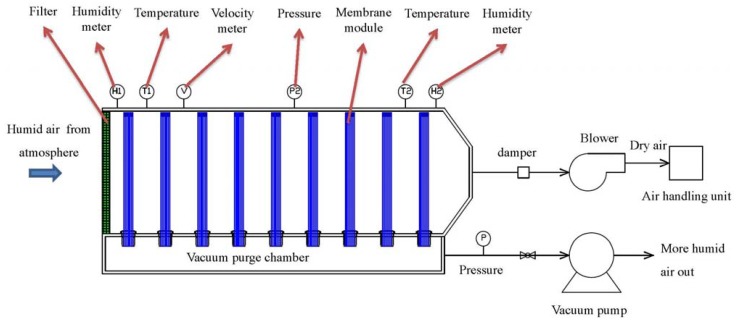
Schematic diagram of membrane dehumidification system.

The membrane dehumidification system is schematically designed in Figure 5. Nine pieces of one-inch caseless fiber bundles were assembled vertically inside the main chamber. One end of the hollow fiber bundles was sealed while the other end was open and connected to the vacuum purge chamber. During the testing, humid outdoor air was drawn into the membrane dehumidification system by a blower at a flow rate of 200 m3/h. While passing the membrane system, the air moisture permeated through the fibers from the shell side to the lumen side. As a result, the dry air was sent to the AHU, whereas the water moisture was removed out of the system by a vacuum at 0.78 bar absolute pressure from the lumen side of the fibers. A vacuum pump with an output of 1.5 kw, a static vacuum pressure of 230 mbar (0.77 bar absolute pressure), the maximum air flow of 3.1 m^3^/min were used. A bypass leaking controlled the actual air flow from the system. Thus, the energy of vacuum was E1 = $\frac{\text{Q}1}{\text{Q}1+\text{Q}2}\;.\text{P}.\text{t}$ = 431.4 KJ where Q1 (permeate air flow from the system) = 5.6 m^3^/h which included the permeating water vapor and air; Q2 (bypass leaking air flow) = 64.5 m^3^/h; P (vacuum pump output) = 1.5 kw; *t* = 3600 s. The pressures, temperatures, and humidity of the inlet and outlet gases were monitored.

## 4. Results and Discussion

### 4.1. The Morphology of PAN Substrates

**Figure 6:**
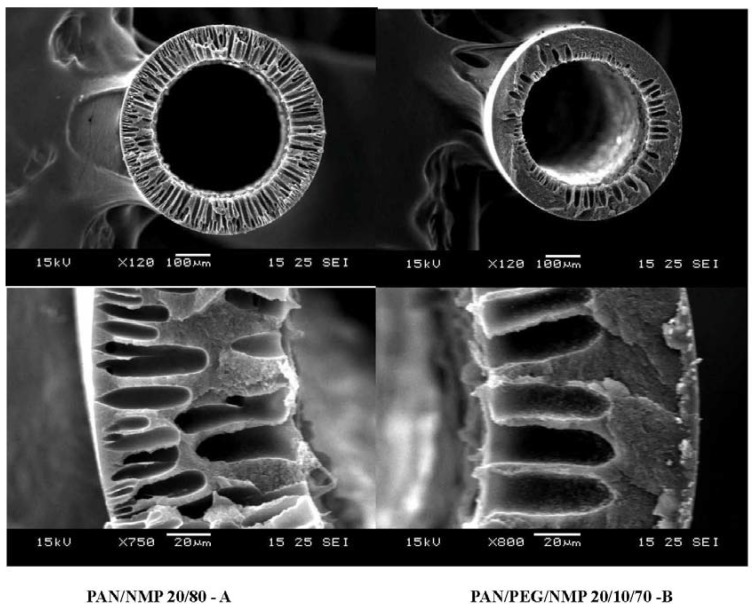
Cross-section morphology of PAN hollow fibers.

To produce PAN/PDMS composite hollow fibers with the maximum performance, several process parameters such as pre-treatment of the PDMS solution, formulation of the dope solution for the PAN substrate, concentration of the coating solution, effect of pre-wetting, and coating time have been studied. The influence of those parameters on fiber performance have been studied systematically and reported by Li *et al.* [25] and Chen *et al.* [43], thus it will not be discussed in the present work. As seen in Table 1, the fiber substrate spun from condition B where PEG was added in the dope formulation has a lower gas permeance possibly because of low cross-section porosity as shown in Figure 6. Therefore, PAN hollow fibers spun from 20/80 PAN/NMP dopes were used for subsequent studies. Table 2 compares the pure gas performance of PAN/PDMS composite hollow fibers as a function of PDMS coating conditions. By using a coating solution of 1% PDMS in hexane, we are able to produce hollow fibers with a CO_2_ permeance of 1766 GPU. A high concentration of PDMS (>1 wt %) leads to an increase in selective layer thickness, which in turn results in a low gas permeance. Since the ideal CO_2_/N_2_ selectivity of all fibers is about the ideal CO_2_/N_2_ selectivity of the PDMS dense film [44], therefore the newly developed fibers are considered to be defect-free.

**Table 1:** Spinning conditions of PAN hollow fibers.

Dope PAN/PEG400/NMP wt %	20/0/80	20/10/70
Spinneret OD/ID	1.6/1.0 mm	
Bore fluid	NMP/Water 90/10 wt %	
External coagulant	water	
Coagulant temperature	25	
Spinning ID	A	B
Dope flow rate (mL/min)	8	8
Bore fluid flow rate (mL/min)	6	6
Air gap (cm)	19	19
Take up speed (m/min)	30	30
O_2_ permeance (GPU)	47,625	31,631
N_2_ permeance (GPU)	52,502	34,025
Selectivity O_2_/N_2_	0.91	0.93

**Table 2:** Coating conditions and pure gas permeation performance of PAN/PDMS fibers.

Hollow Fiber Substrate		PAN-20/80				
ID of Modules		#1	#2	#3	#4	#5
Gas Permeation Performance of the PAN Substrate (No Coating)						
Gas permeance (GPU)	O_2_	47,625	47,625	47,625	47,625	47,625
	N_2_	52,502	52,502	52,502	52,502	52,502
	CO_2_	43,065	43,065	43,065	43,065	43,065
Concentration of PDMS in Hexane		1%	2%	3%	4%	5%
Pre-wetting		No				
Coating time		1 s	1 s	1 s	1 s	1 s
Gas permeance (GPU)	O_2_	321	180	182	124	90
	N_2_	143	81	84	55	29
	CO_2_	1766	1075	1073	728	375
Selectivity	O_2_/N_2_	2.3	2.2	2.2	2.2	3.1
	CO_2_/N_2_	12.4	13.2	12.8	13.2	12.9

### 4.2. Pure Gas Permeance and Dehumidification Performance of One-inch Hollow Fiber Modules

Table 3 summarizes the pure gas and water vapor performance of nine pieces of one-inch PAN/PDMS hollow fiber modules. The results show that both the gas permeance and the ideal selectivity of the one-inch bundles are lower than those of single fibers as shown in Table 2. The lower gas permeance is possibly caused by two reasons. Firstly, the PDMS coating is a very sensitive process. During the batch coating of 400 pieces of fibers at the same time, the PDMS coating may not be as effective and homogeneous as compared to a single fiber coating. Secondly, some membrane areas may not be effectively used during the separation (so-called the dead area). The low gas pair selectivity is mainly due to the fact that the batch coating of 400 pieces of fibers is not as efficient and some fibers might be defective. The defective fibers can be repaired by improving the coating precision [45]. However, the process might be complicated, time consuming and expensive. According to Rice and Murphy’s analyses [46], the membranes need not be defect-free to achieve an efficient dehumidification. As long as the membranes have an O_2_/N_2_ selectivity of around 1–2, water moisture can still be effectively removed. The testing results of our pilot in the following Section 4.3 will further validate this statement.

**Table 3:** Pure gas and water vapor permeation performance of 9 pieces of one-inch PAN/PDMS hollow fiber modules.

ID of Module	#1	#2	#3	#4	#5	#6	#7	#8	#9
N_2_ permeance (GPU)	141	79	51	328	161	188	73	246	67
O_2_ permeance (GPU)	205	94	82	400	225	329	123	376	100
CO_2_ permeance (GPU)	740	197	273	907	311	786	164	612	184
H_2_O permeance (GPU)	12,827	10,781	11,227	11,345	11,041	12,707	9011	12,547	8647
Selectivity O_2_/N_2_	1.5	1.2	1.6	1.2	1.4	1.8	1.7	1.5	1.5
Selectivity CO_2_/N_2_	5.3	2.5	5.4	2.8	1.9	4.2	2.2	2.5	2.7
Selectivity H_2_O/N_2_	91.1	137.1	220.2	34.6	68.8	67.6	122.8	51.1	128.6

The pure gas permeation performance was tested by single gas while water vapor permeation performance tested by outdoor humid air.

### 4.3. Membrane Dehumidification Field Tests

**Figure 7:**
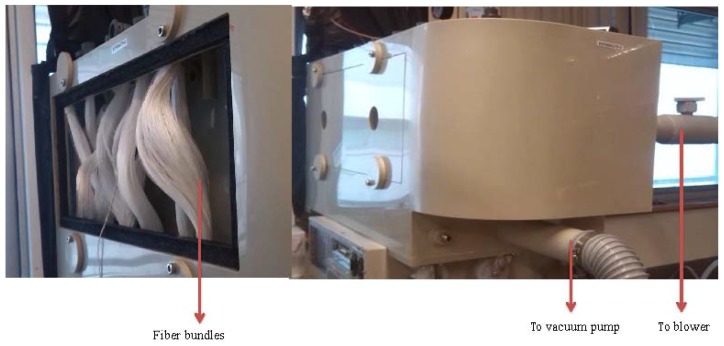
Home-made fiber bundles and the dehumidification pilot unit.

To achieve a reasonable gas flux to feed into AHU, more than one piece of hollow fiber modules are required (where the number of hollow fiber modules can be calculated by knowing the effective gas flux per module and the required gas flow by the AHU). When multiple modules are used, they are often connected in parallel or in series. With the objectives to (1) reduce the cost of PVC materials and connection accessories, (2) simplify the module connection, as well as (3) reduce the gas pressure loss inside the connection passages, one-inch hollow fiber bundles without housing and a system comprising nine pieces of one-inch hollow fiber bundles are taken as our design. Figure 7 shows the pictures of our membrane dehumidification system.

**Figure 8:**
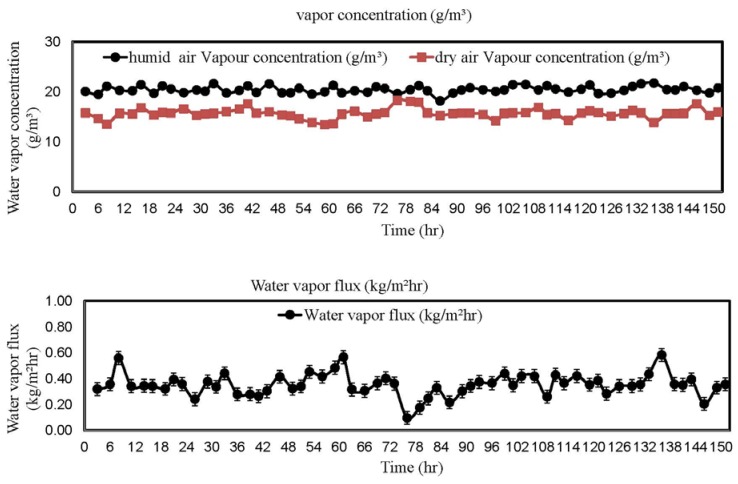
Water vapor concentration and flux *vs*. time.

The dehumidification pilot was tested in National University of Singapore. During the test, the feed side of the pilot was located outside the window where fresh outdoor air was directly drawn into the membrane system by a blower. A continuous 150 h test was carried out and the results are plotted in Figure 8. In our test, the inlet fresh air has an average water vapor concentration of 20 ± 2 g/m. After dehumidification, the dry air has an average water vapor concentration of 15.9 ± 2.4 g/m. In other words, about 20.5% of water vapor concentration can be effectively removed by our dehumidification system. This result supports our previous statement that an effective membrane dehumidifier does not require membranes with a high selectivity, but the membranes must have a reasonably high gas permeance. Another interesting observation is the dehumidification performance is stable along the 150 h test.

### 4.4. Calculations on Energy Consumption

**Figure 9:**
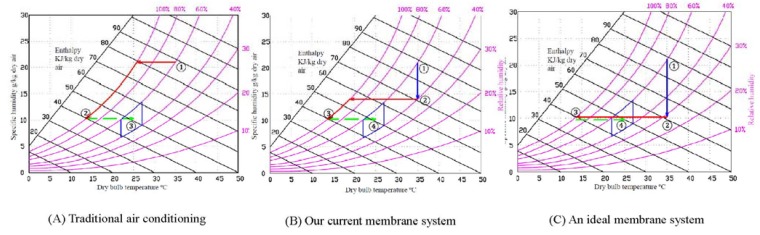
Comparison of energy consumption of (**A**) traditional air conditioning system without dehumidification; (**B**) air conditioning using our current membrane system; and (**C**) air conditioning using an ideal membrane system.

With the aid of the psychrometric chart [42] in Figure 9, the energy consumption of different cooling approaches are calculated using the following equations:

(5) $$E=\frac{q.Q.\rho .t}{C}$$

(6) $$q=h_{1}-h_{2}$$

where E = Energy consumed in total (KJ),

q = Heat removed (KJ/kg dry air),

h_1_ = Enthalpy of air (KJ/kg dry air) at point 1,

h_2_ = Enthalpy of air (KJ/kg dry air) at point 2,

Q = Air flow rate (m^3^/h),

ρ = Air density (kg/m^3^),

C = Coefficient of performance (refrigerator),

t = period of time (s)

W_1_ = Specific humidity (g/kg dry air) at point 1,

W_2_ = Specific humidity (g/kg dry air) at point 2,

φ_1_ = Relative humidity at point 1,

φ_2_ = Relative humidity at point 2

In the following calculations, it is assumed that the temperature and relative humidity of the feed gas is 35 °C and 60%, respectively, while the air flow rate Q = 200 m^3^/h.

In a traditional air conditioning (Figure 9A), the humid hot outdoor air is directly compressed and cooled down to around 15 °C. To reach the comfort zone for human beings in the indoor environment, the air flow is usually mixed up with hot outdoor air to reach 22 to 27 °C. From the chart, it is read that W_1_ = 21.75 g H_2_O/kg-dry-air and W_2_ = 10.25 g H_2_O/kg-dry-air; hence, the amount of moisture removed from W_1_ to W_2_ is 11.5 g H_2_O/kg-dry-air. The enthalpy h_1_ = 88 KJ/kg-dry-air and h_2_ = 40 KJ/kg-dry-air. Assuming the chiller firstly cools the air to 15 °C, q_cool_ = h_1_ − h_2_ = 48 kJ/kg dry air, the coefficient of performance C = 4, *t* = 3600 s. The energy to mix up the hot air is ignored. The energy consumed for this traditional process is mainly the energy for cooling (*i.e*., E = 2795 KJ).

In the ideal case, the humidity of the outdoor can be reduced by a membrane dehumidification system from about 60% to 30% and after that the dried hot air is cooled to 15 °C. The pathway is demonstrated in Figure 9C. From point 1 (60%) to point 2 (30%), the energy consumed is mainly from the energy of vacuum, E_1_ = $\frac{\text{Q}1}{\text{Q}1+\text{Q}2}\;.P.t$ = 431.4 KJ where Q1 (permeate air flow from the system) = 5.6 m^3^/h; Q2 (bypass leaking air flow) = 64.5 m^3^/h; P (vacuum pump output) = 1.5 kw; *t* = 3600 s. To cool the air from 35 °C to 15 °C, the enthalpy h_2_ = 60 KJ/kg-dry-air, h_3_ = 40 KJ/kg-dry-air, q_cool_ = h_2_ − h_3_ = 20 kJ/kg dry air. The energy consumed for cooling E_2_ = 1165 KJ. Thus, the total energy consumed is equal to E = E_1_ + E_2_ = 1596.4 KJ. Compared to the conventional air conditioning, using an ideal membrane dehumidifier can save the energy of 42.9%.

Using our current membrane dehumidification pilot, the humidity of the outdoor air is firstly removed to about 40% to 50%. After that, the relatively dried air is cooled down to around 15 °C. Similarly to Figure 9A, the air flow is usually mixed up with outdoor hot air before it is circulated to indoor. The pathway is demonstrated in Figure 9B. This is because our membrane performance and system design has not been fully optimized. 40% is the best RH that can be achieved by our current membrane system.

For the chart, it is read that W_1_ = 21.75 g H_2_O/kg-dry-air, W_2_ = 14.25 g H_2_O/kg-dry-air, and W_3_ = 10.25 g H_2_O/kg-dry-air, the amount of moisture removed from W_1_ to W_2_ is 7.5 g H_2_O/kg-dry-air. During this process, the energy consumed is merely vacuum which is costed by the vacuum pump the same as the ideal case. Hence, the energy consumed in 1 h for dehumidification E_1_ = 431.4 KJ. Since h_2_ = 68 KJ/kg-dry-air, h_3_ = 40 KJ/kg-dry-air, q_cool_ = h_2_ − h_3_ = 28 kJ/kg dry air, *t* = 3600 s. The energy consumed for cooling E_2_ = 1630.3 KJ. Thus, the total energy consumed is equal to E = E_1_ + E_2_ = 2061.7 KJ. In conclusion, the combined use of our membrane dehumidifier together with air conditioning can reduce energy consumption by up to 26.2% compared to conventional air conditioning processes.

## 5. State-of-Art on Membrane Dehumidification and Conclusions

There are commercial products available on the market for gas separation membranes and membrane modules. Our composite PAN/PDMS membrane has the advantage of cheaper materials costs as compared to polysulfone (PSF) fibers made by Air Product US or Generon UK. Moreover, the newly developed fibers can be operated at a lower pressure with reasonably good performance. Compared to the PDMS fibers made by MedArray via melt spinning, our approach is less energy intensive, specifically during the fabrication process. Using our current hollow fiber membranes and membrane dehumidification system, the energy saving for air conditioning is up to 26.2%. With further improvements of our membranes and membrane modules’ performance, as well as the optimization of the membrane system design, the energy saving of our membrane dehumidifier will be further improved.
